# ZB-16, a Novel GPR119 Agonist, Relieves the Severity of Streptozotocin–Nicotinamide-Induced Diabetes in Rats

**DOI:** 10.3389/fendo.2017.00152

**Published:** 2017-07-07

**Authors:** Ivan N. Tyurenkov, Denis V. Kurkin, Dmitry A. Bakulin, Elena V. Volotova, Mikhail A. Chafeev, Alexey V. Smirnov, Evgeny I. Morkovin

**Affiliations:** ^1^Volgograd State Medical University (VSMU), Volgograd, Russia; ^2^Chemical Diversity Research Institute, Khimki, Russia; ^3^Volgograd Medical Research Center (VMRC), Volgograd, Russia

**Keywords:** GPR119, ZB-16, diabetes mellitus, rodents, streptozotocin–nicotinamide diabetes

## Abstract

GPR119 is involved in the regulation of incretin and insulin secretion, so the GPR119 agonists have been suggested as novel antidiabetic medications. The purpose of this work was to assess the influence of novel GPR119 agonist ZB-16 on the glucose utilization, insulin, and glucagon-like peptide-1 (GLP-1) secretion and the morphology of pancreas in rats with streptozotocin–nicotinamide-induced diabetes. 45 male Wistar rats were used in the study. The criteria of streptozotocin–nicotinamide-induced diabetes were blood glucose levels of 9–14 mmol/l measured in fasting conditions on the third day since administration of streptozotocin (65 mg/kg) and nicotinamide (230 mg/kg). Animals failed to reach the criteria were excluded from the experiment. The substances were administered *per os* once per day for 28 days. Measurements included blood glucose monitoring (every 7 days), glucose tolerance test (every 14 days), the assessment of insulin and GLP-1 levels in blood plasma (28 days after beginning), and the results of immunohistochemical staining of pancreas. It was found that ZB-16 (1 mg/kg *per os*, once a day) decreases the blood glucose levels under fasting conditions and improves the glucose utilization. These changes were associated with the increase in stimulated secretion of GLP-1 and insulin, accompanied by the growth of insulin-positive cells in pancreas. Thus, ZB-16 could be a promising antidiabetic drug for oral administration.

## Introduction

Incretins are metabolic hormones which decrease the blood glucose level. This effect is provided by the increase in the amount of insulin released from pancreatic β-cells. They also slow down the rate of absorption of nutrients into the blood stream by reducing gastric emptying and may directly reduce food intake. They also inhibit glucagon release from the alpha cells of the islets of Langerhans. Furthermore, it is worth mentioning that incretins [GIP and glucagon-like peptide-1 (GLP-1)] slow down the apoptosis of pancreatic β-cells, induce reparation and growth of pancreatic islets (1).

Medications manipulating the incretin system are one of the main drugs that are currently used to treat type 2 diabetes mellitus. GLP-1, one of the main candidate molecules, is rapidly inactivated by the enzyme dipeptidyl peptidase-4 (DPP-4). This requires the continuous subcutaneous infusion, which is not convenient for patients. Drugs acting *via* the incretin system are DPP-4 inhibitors and GLP-1 receptor agonists that are more resistant to enzymatic degradation. Short lifetime of native GLP-1 was a reason for the development of several long-lasting analogs with twice daily (exenatide), once daily (lixisenatide and liraglutide), and once weekly (exenatide ER, albiglutide, and dulaglutide) injection that have been approved for use in the US. GLP-1 receptor agonists are effective enough; however, the main disadvantage of these medications is that they have only injectable forms that are less convenient than oral administration for patients with type 2 diabetes mellitus (2). Alternative approach is to inhibit the enzyme DPP-4 that inactivates native GLP-1. Over the past 10 years, more than 10 DPP-4 inhibitors were approved for use in different countries (3).

Since a number of specific receptors (GPR40, GPR41, GPR43, GPR119, and GPR120) expressed on the enteroendocrine L- and K-cells were discovered, there has been an increasing interest in making new effective drugs to treat type 2 diabetes mellitus and metabolic syndrome (4). Leading pharmaceutical companies (Arena Pharmaceuticals, GlaxoSmithKline, Sanofi, Astellas, etc.) are searching for agonists of these receptors. The main characteristic of these drugs is their ability to increase incretins and insulin secretion in response to food intake, which is important to maintain the effective control of postprandial blood glucose level.

From this point, the aim of the current work was to assess the effects of novel GPR119 agonist ZB-16 including the influence of the substance on the glucose utilization, insulin, and GLP-1 secretion and the morphology of pancreas during the chronic administration to the rats with streptozotocin–nicotinamide-induced diabetes.

## Materials and Methods

### Materials

**Figure 1:**
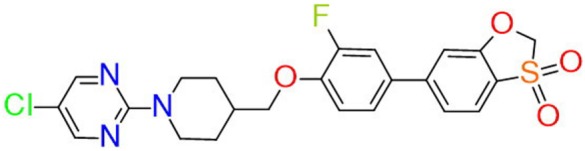
Chemical structure of ZB-16.

The synthesis of a new GPR119 agonist ZB-16 was performed by Chemical Diversity Research Institute (Khimki, Russian Federation). The structure of ZB-16 is shown in Figure 1.

The specific activity of ZB-16 (previously ZB40-0016) on GPR119 was tested previously as described at RUS Patent 2576037 C1, Russia (2014) (5). Briefly, CHO-K1 cell line expressing human GPR119 (hGPR119) receptors was used. The agonistic activity of the ZB-16 was estimated by the increase in intracellular cAMP concentration, as it is known that, when activated, hGPR119 receptors cause subsequent activation of cellular adenylate cyclase, which increases intracellular cAMP levels (6). Lance Ultra cAMP kit (Perkin Elmer, Waltham, MA, USA) was chosen as the experimental platform. Known agonists of hGPR119 receptors were used as positive controls and to determine the upper limit (maximal signal) of the experiment (see Figure S1 in Supplementary Material).

It was found that the following activation of GPR119 leads to the increase in intracellular cAMP at nanomolar concentrations of ZB-16 (EC50 = 7.25 × 10^−9^ M). ZB-16 also demonstrated low toxicity and the lack of inhibitory effects on CYP450 isoforms at HepG2 cell line. Preliminary *in vivo* pharmacokinetic studies were also performed (*T*_max_ = 4 h, *T*_1/2_ = 13 h) at a dose of 10 mg/kg *per os* (7).

Streptozotocin and nicotinamide were purchased from Sigma-Aldrich (USA). Rat insulin ELISA kit was purchased from Mercodia (Sweden). Rat GLP-1 ELISA kit was purchased from Wuhan (China). Other reagents and solvents were of first grade and commercially available.

### Animals

Animal experiments were conducted in accordance with animal research standards defined by Russian law and EASC technical standards for Good laboratory practice (GOST R 53434-2009 and GOST R 51000.4-2011). The study design and the protocol were reviewed and approved by the body responsible for providing ethics approval—Department of the ethical, legal, and sociological expertise in medicine of the Volgograd Medical Research Center [registration number: IRB 00005839 IORG 0004900 (OHRP)] on February 2, 2014 (protocol number 191-2014).

45 male Wistar rats weighing 280–330 g (5–6 months) bred in the federal laboratory animal house “Rappolovo” (Leningrad Oblast, Russian Federation) were used in this study. Animals were acclimated for 14 days before starting the experiment. They were housed at 20 ± 2°C and 40–60% humidity in a standard 12/12-h light–dark cycle with food and tap water *ad libitum*. Before some tests described below the animals were fasted overnight (8 h) with full access to tap water. All manipulations listed below were performed at the same time to prevent the additional stress decreasing the habituation in animals.

**Figure 2:**
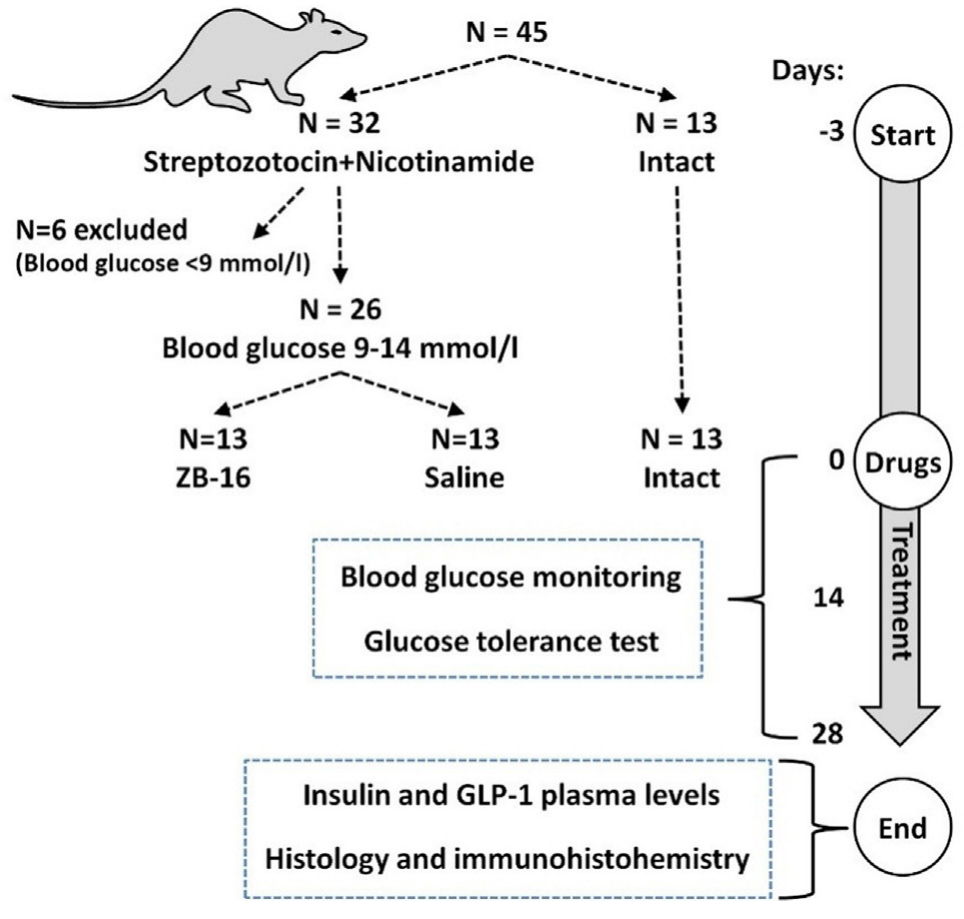
Experimental timeline and animal usage.

The whole experimental design is demonstrated in Figure 2. The induction of experimental diabetes mellitus in 32 animals is described below. Animals with streptozotocin–nicotinamide-induced diabetes (*N* = 26) were randomly divided into two groups: the control group (saline, 5 ml/kg *per os*) and the experimental group (ZB-16, 1 mg/kg, dissolved in saline, 5 ml/kg *per os*), six animals were excluded from the experiment. The administration was performed *via* intragastral gavage once a day at the morning (1 h after lights on). The intact group of 13 healthy animals habituated to the same regiment and gavaging (saline, 5 ml/kg *per os*) was also included in this study.

### Streptozotocin–Nicotinamide Diabetes (StD) Modeling

The StD is a highly reproducible animal model of diabetes mellitus (8, 9). Streptozotocin demonstrates a cytotoxic action on pancreatic B-cells, whereas nicotinamide partially protects these cells against streptozotocin. This combination provides the most common symptoms of diabetes mellitus including high blood glucose levels and glucose tolerance disturbances. Procedures were performed in 32 animals. Streptozotocin (65 mg/kg) was administered i.p. to overnight fasted rats in 100 mmol/l citrate buffer (pH = 4.5). Nicotinamide (230 mg/kg) dissolved in saline was administered i.p. 15 min before streptozotocin. The criteria of streptozotocin–nicotinamide-induced diabetes were blood glucose levels 9–14 mmol/l measured in fasting conditions on the third day since the administration of streptozotocin and nicotinamide. Animals failed to reach the criteria (*N* = 6) were excluded from the current experiment.

### Biochemical Analysis

Blood glucose levels were measured with the blood glucose meter Contour TS (Bayer Diabetes Care, UK) after the collection of blood samples from the tail vein.

The oral glucose tolerance test was performed on the 14th and 28th day of the current study. Overnight fasted animals 2 h after drug or saline administration were given 3 g/kg of 40% glucose solution *via* intragastral gavage. Blood glucose levels were measured before and 30, 60, and 120 min after the administration of glucose solution. Area under the curve was calculated *via* GraphPad Prism 5.0.

Blood samples for measuring insulin and GLP-1 plasma levels were collected twice: before the glucose tolerance test and 15 min after the administration of glucose solution. Microcentrifuge tubes, containing heparinized blood samples, were centrifuged at 3,000 rpm for 15 min. The supernatant was sampled and stored at −20°C until the assay.

Plasma levels of insulin and GLP-1 were assessed using “Mercodia Rat Insulin ELISA Kit” (Mercodia, Sweden) and “Rat glucagon-like peptide-1 (GLP-1) ELISA Kit” (Wuhan, China), correspondingly.

### Histology and Immunohistochemistry

Animals were sacrificed *via* decapitation at the end of the experiment (animals were anesthetized with chloral hydrate, 400 mg/kg i.p.). The entire pancreas was analyzed, which was divided into three parts: intestinal, gastric, and splenic. From each part, four slices (12 slices per animal) were made: two (paraffin slices, 4 µm) were stained with hematoxylin and eosin and another two used for immunohistochemical staining [with murine monoclonal antibodies to insulin (1G4 clone, GeneTex) diluted to 1:100]. EnVision system (Thermo Scientific, Fremont, CA, USA) was used for visualization with diaminobenzidine (Thermo Scientific, Fremont, CA, USA) as chromogenic substrate. After the immunological reaction tissues were stained with hematoxylin. Imaging was performed with the microscope Axiostar plus (Germany) and digital camera Canon (Japan). Morphometric assay of pancreatic islets included the measurement of the average islet area (*S*, μm^2^), average islet perimeter (*L*, μm), average area of insulin-positive endocrine cells (*S*, μm^2^), and average relative area of insulin-positive endocrine cells (the ratio of the area of insulin-positive material to the area of the island, %).

### Statistical Analysis

Statistical analysis was performed using the following software: Microsoft Office Excel 2013 (Microsoft, USA), Statistica 8.0 (Statsoft, Inc., USA), and GraphPad Prism 5.0 (GraphPad Software, Inc., USA). The distribution of all data was estimated using the Shapiro–Wilk normality test. Kruskal–Wallis test with Dunn’s *post hoc* test was used for non-parametric data distribution. One-way ANOVA or repeated measures two-way ANOVA with Newman–Keuls *post hoc* test was performed for Gaussian data sets (indicated in the figure legends).

## Results

### The Assessment of the Hypoglycemic Action of the GPR119 Receptor Agonist in Animals with Type 2 Diabetes Mellitus

The GPR119 agonists are potent antidiabetic compounds with the incretin-like type of action. They could be used for long-term oral administration in type 2 diabetes mellitus patients either in monotherapy or, depending on the severity of diabetes, in combination with the other oral hypoglycemic drugs.

Based on the previous studies of ZB-16 (7), this stage aims to estimate the hypoglycemic action of the GPR119 receptor agonist in animals with type 2 diabetes mellitus during a 28-day treatment.

**Figure 3:**
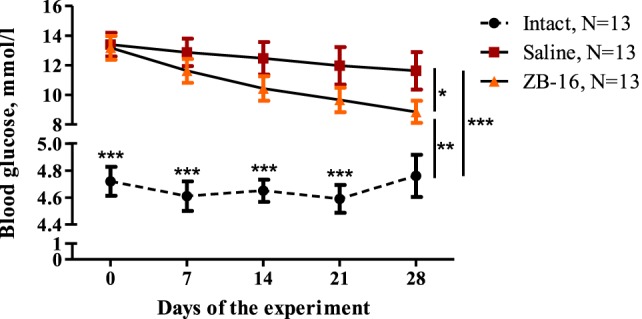
The blood glucose dynamics during the experiment. Blood glucose levels measured under fasting conditions every week from the beginning of the experiment are shown as M ± SEM. Black circles—intact group of healthy animals, receiving saline (5 ml/kg *per os*); red rectangles—control group with streptozotocin–nicotinamide diabetes (StD), receiving saline (5 ml/kg *per os*); and orange triangles—experimental group with StD, receiving the novel GPR119 agonist ZB-16 (1 mg/kg, dissolved in 5 ml/kg of saline, *per os*). All procedures were performed once a day at the morning. Repeated measures two-way ANOVA: time—*p* = 0.029; treatment—*p* < 0.0001; time × treatment—*p* = 0.401. Newman–Keuls *post hoc* test: for intact group *p* < 0.001 both with control and ZB-16 groups from 0th to 21st day of experiment; 28th day: *p < 0.05, **p < 0.01, and ***p < 0.001 (comprised data sets are shown with vertical lines).

On the third day of streptozotocin and nicotinamide administration, the blood glucose levels in experimental animals increased significantly (12.7–13.4 mmol/l in the experimental group vs. 4.0–4.5 mmol/l in intact animals). During the therapy, the glucose levels in fasting conditions tended to decline both in the saline and ZB-16 group, but the difference between them became significant (*p* < 0.05) on the 28th day of the experiment (11.6 vs. 8.9 mmol/l) (Figure 3).

**Figure 4:**
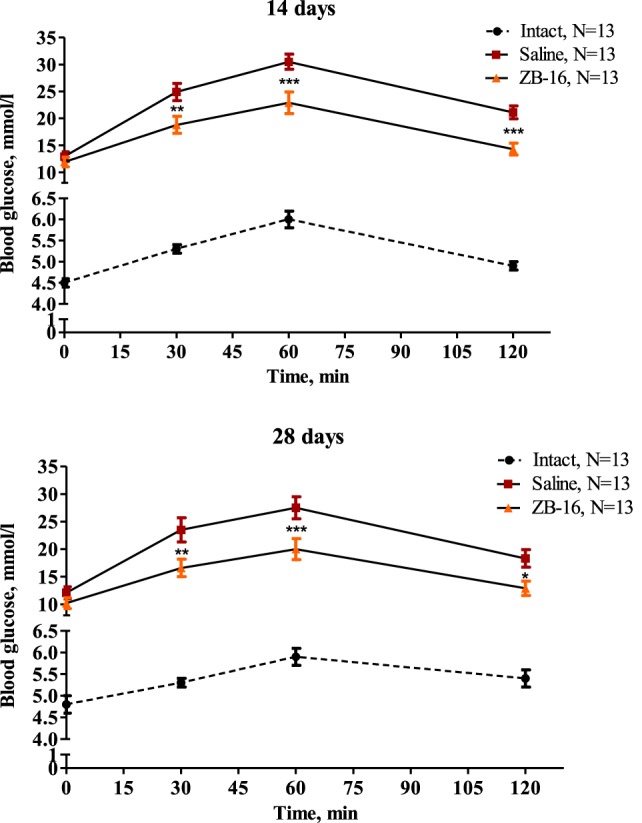
Glucose tolerance test performed on the 14th and 28th day of the experiment. Blood glucose levels measured during the oral glucose tolerance test on 14th and 28th day of the experiment are shown as M ± SEM. The test was performed on overnight fasted animals given 3 g/kg of 40% glucose solution *via* intragastral gavage. Black circles—intact group of healthy animals, receiving saline (5 ml/kg *per os*); red rectangles—control group with streptozotocin–nicotinamide diabetes (StD), receiving saline (5 ml/kg *per os*); and orange triangles—experimental group with StD, receiving the novel GPR119 agonist ZB-16 (1 mg/kg, dissolved in 5 ml/kg of saline, *per os*). Repeated measures two-way ANOVA: time—*p* < 0.0001; treatment—*p* < 0.0001; time × treatment—*p* < 0.0001 for both testing days. Newman–Keuls *post hoc* test: *p < 0.05, **p < 0.01, and ***p < 0.001 between saline and ZB-16 groups; and for intact group *p* < 0.001 both with control and ZB-16 groups in all time points.

**Figure 5:**
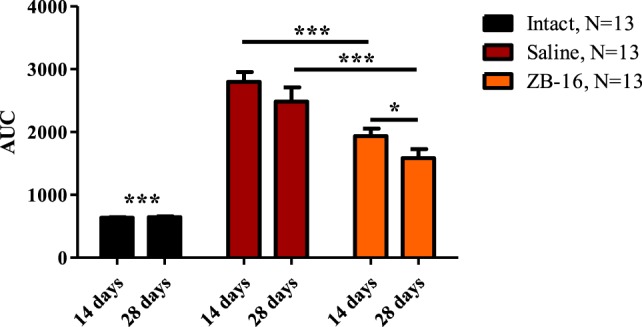
Area under the curve “Blood glucose vs. Time” during the glucose tolerance test. The figure demonstrates the area under the curve “blood glucose/time” plotted during the oral glucose tolerance test on 14th and 28th day of the experiment. Data are shown as M ± SEM. The test was performed on overnight fasted animals given 3 g/kg of 40% glucose solution *via* intragastral gavage. Black—intact group of healthy animals, receiving saline (5 ml/kg *per os*); red—control group with streptozotocin–nicotinamide diabetes (StD), receiving saline (5 ml/kg *per os*); orange—experimental group with StD, receiving the novel GPR119 agonist ZB-16 (1 mg/kg, dissolved in 5 ml/kg of saline, *per os*). Two-way ANOVA: time—*p* = 0.0018; treatment—*p* < 0.0001; time × treatment—*p* = 0.0629. Newman–Keuls *post hoc* test: *p < 0.05, ***p < 0.001 (comprised data sets are shown with horizontal lines); for intact group *p* < 0.001 both with control and ZB-16 groups.

During the first 14 days, fasting blood glucose levels reached 12.9 ± 0.9 mmol/l in the saline-treated group vs. 11.9 ± 0.9 mmol/l in the ZB-16 group with the absence of significant difference between the groups. However, the glucose tolerance test showed that there was significant retardation of blood glucose increase in the ZB-16 group either on the 14th or 28th day of the experiment (Figure 4). These changes were associated with the decrease in AUC “Blood glucose vs. Time,” which was also statistically significant (Figure 5). Thus, the new GPR119 agonist ZB-16 demonstrated the mild hypoglycemic activity on glucose-loaded animals with the experimental type 2 diabetes mellitus caused by streptozotocin and nicotinamide administration.

### The Effects of GPR119 Agonist ZB-16 on GLP-1 and Insulin Concentrations in Blood Plasma after the Glucose Load in Animals with Type 2 Diabetes Mellitus on Day 28

This stage of the current study was to assess the effects of ZB-16 on the secretion of incretins and insulin, the well-known action mechanism of GPR119 agonists (4). The insulin secretion undergoes several stages following the GLP-1 secretion. GLP-1 levels reach maximum secretion 15–20 min after oral glucose administration, followed by a slow decline toward fasting levels (9, 10). From this point, we decided to assay the GLP-1 and insulin on 15 min after the glucose administration during the glucose tolerance test described above.

**Figure 6:**
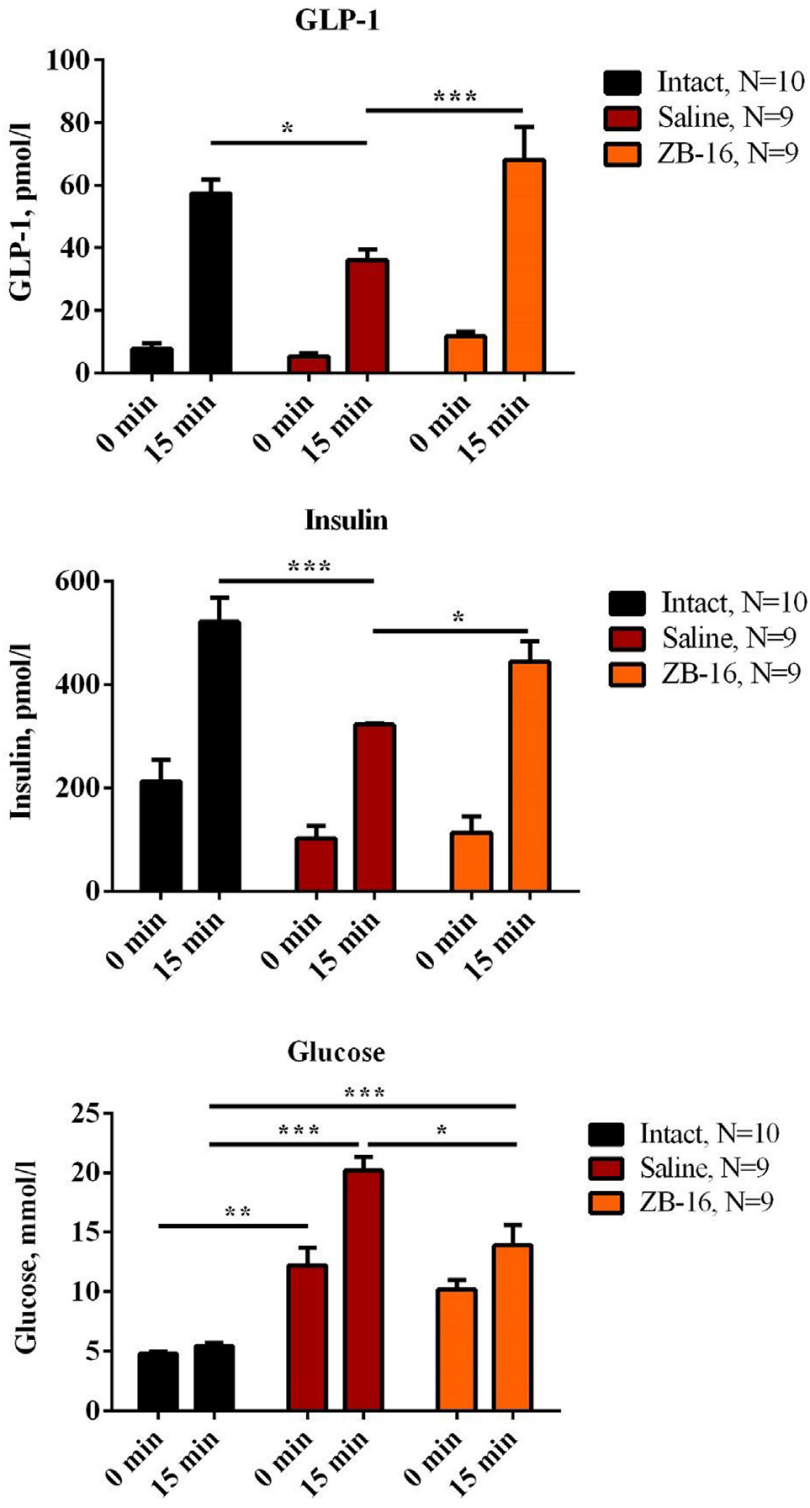
The effects of GPR119 agonist ZB-16 on glucagon-like peptide-1 (GLP-1) and insulin concentrations after the glucose load on the 28th day of experiment. The figure demonstrates the blood plasma concentrations of GLP-1 and insulin measured on 28th day of experiment during the oral glucose tolerance test. Data are shown as M ± SEM. The test was performed on overnight fasted animals given 3 g/kg of 40% glucose solution *via* intragastral gavage. Black—intact group of healthy animals, receiving saline (5 ml/kg *per os*); red—control group with streptozotocin–nicotinamide diabetes (StD), receiving saline (5 ml/kg *per os*); orange—experimental group with StD, receiving the novel GPR119 agonist ZB-16 (1 mg/kg, dissolved in 5 ml/kg of saline, *per os*). Two-way ANOVA: GLP-1: time—*p* < 0.0001; treatment—*p* = 0.0060; and time × treatment—*p* = 0.0310. Newman–Keuls *post hoc* test: *p < 0.05, ***p < 0.001 (comprised data sets are shown with horizontal lines). Insulin: time—*p* < 0.0001; treatment—*p* = 0.0043; and time × treatment—*p* = 0.0870. Newman–Keuls *post hoc* test: *p < 0.05, ***p < 0.001 (comprised data sets are shown with horizontal lines). Blood glucose: time—*p* < 0.0001; treatment—*p* < 0.0001; and time × treatment—*p* = 0.0002. Newman–Keuls *post hoc* test: *p < 0.05, **p < 0.01, and ***p < 0.001 (comprised data sets are shown with horizontal lines).

The initial levels of GLP-1 were almost equal in the intact and both experimental groups (Figure 6). The glucose administration resulted in the increase in GLP-1 plasma levels, which became significantly lower in the saline-treated group in comparison to the intact group (*p* < 0.01) and the ZB-16-treated group (*p* < 0.001). The insulin secretion (Figure 6) changed similarly with the lowest levels found in the saline-treated group. We found the absence of statistically significant differences in GLP-1 and insulin secretion between the intact and ZB-16 groups. Thus, ZB-16 rescues the stimulated insulin and incretin secretion in animals with type 2 diabetes mellitus caused by streptozotocin and nicotinamide administration.

### Histological Assay of the Insulin Expression

**Figure 7:**
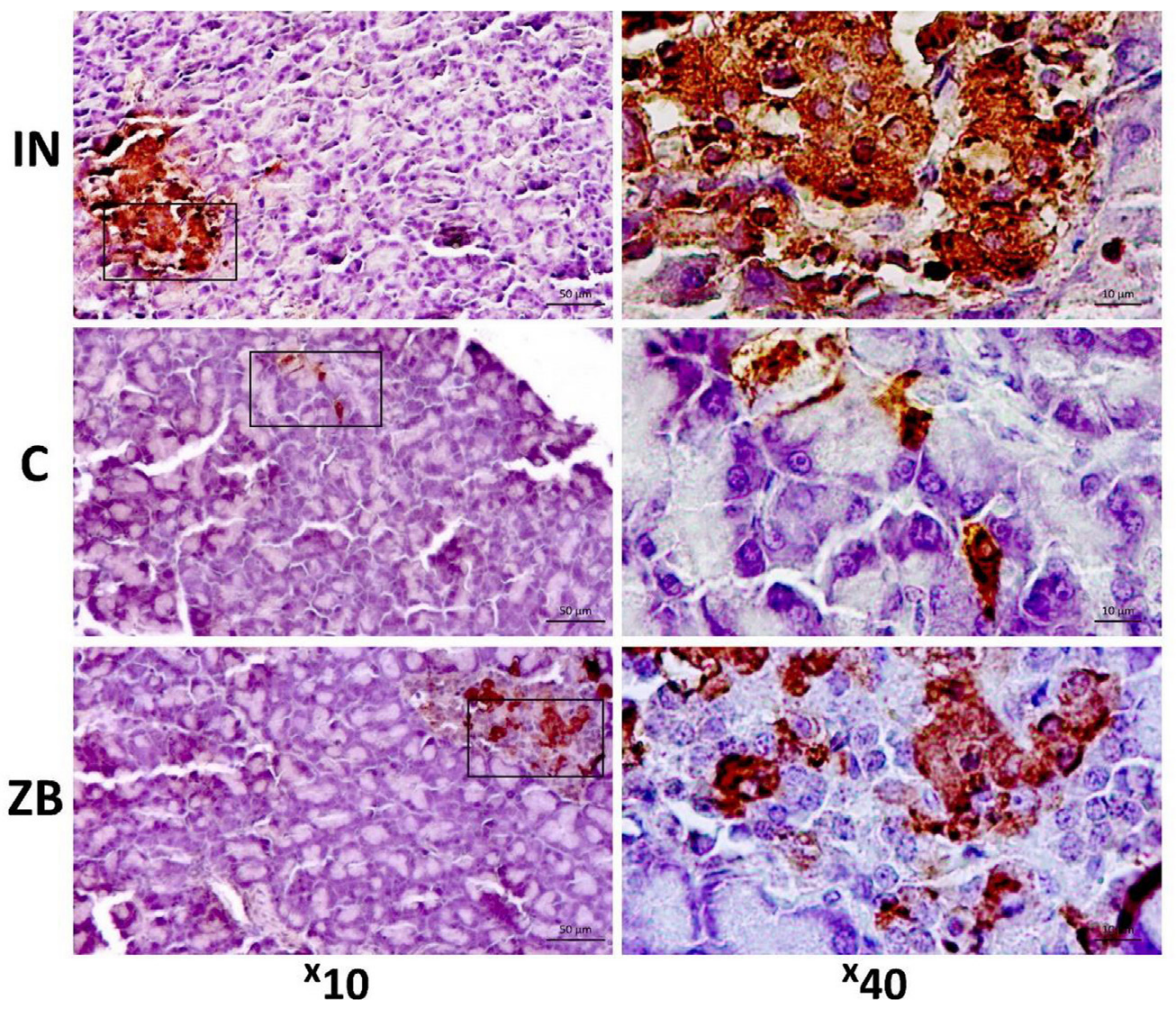
Morphological changes in pancreatic islets. The tissue sections were stained with murine monoclonal antibodies to insulin with diaminobenzidine as chromogenic substrate, additional staining was performed with hematoxylin. Intact (IN)—intact group of healthy animals; the prevalence of insulin-positive β-endocrine cells. Control (C)—saline-treated group with experimental type 2 diabetes mellitus; the decrease in insulin-positive material. ZB—group of animals treated with ZB-16; the moderate increase in β-endocrine cells in comparison to the saline-treated group.

The morphological changes found in the saline-treated group include the focal hyperemia of pancreas (Figure 7) associated with the focal lymphoid infiltration of the intralobular connective tissue or the interstitial edema in some cases. The endocrine cells and their nuclei were hypertrophied. The periphery of pancreatic islets was partially infiltrated with lymphocytes. The pancreatic islets had irregular shapes and contours, their size and number decreased dramatically in comparison to those of the intact group (Figure 7). The hypoglycemic action of ZB-16, described above, could be connected with the rescue of pancreatic β-cells as minor pathological changes were observed in the pancreas (Figure 7).

**Figure 8:**
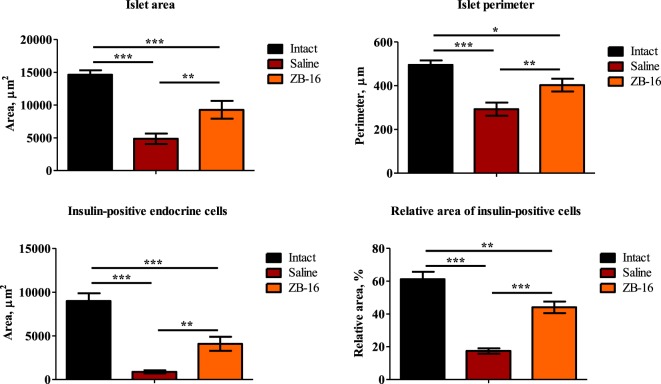
The morphometric changes in pancreatic islets. The figure shows the morphometric assay results performed after 28 days of treatment. Top: left—islet area, μm^2^; right—islet perimeter, μm^2^; bottom: left—area of insulin-positive endocrine cells, μm^2^; right—relative area of insulin-positive endocrine cells, %. All data shown as M ± SEM. Black—intact group of healthy animals, receiving saline (5 ml/kg *per os*); red—control group with streptozotocin–nicotinamide diabetes (StD), receiving saline (5 ml/kg *per os*); orange—experimental group with StD, receiving the novel GPR119 agonist ZB-16 (1 mg/kg, dissolved in 5 ml/kg of saline, *per os*). One-way ANOVA, Newman–Keuls *post hoc* test: *p < 0.01, **p < 0.01, and ***p < 0.001 (comprised data sets are shown with horizontal lines).

The results of the morphometric assay presented in Figure 8 show that the size and the area of pancreatic islets in animals with experimental diabetes declined significantly in comparison to the intact group (Figure 8). This could be interpreted as the typical result of streptozotocin-induced diabetes mellitus (11). However, the significant differences were observed between the saline-treated and ZB-16 groups. It was found that the administration of the new GPR119 agonist leads to the proliferation of insulin-positive endocrine cells, which results in the recovery of the pancreatic islets. Since the size of β-endocrine cells was comparable with the dimensions of similar cells in the control group, it is assumed that the increase in the number of β-endocrine cells occurs due to the proliferation and the differentiation of cambial cells reducing the severity of atrophic changes.

## Discussion

Compound ZB-16 being administrated *per os* on a regular basis has a significant antidiabetic activity connected with the increase in glucose-stimulated insulin secretion. Although ZB-16 demonstrated the absence of significant influence on fasting plasma glucose levels, the significant increase in the glucose utilization during the glucose tolerance test had been found. Our findings match those observed in earlier studies (12). Previously in comparable studies was shown that hypoglycemic activity of ZB-16 (1 mg/kg) in rats with streptozotocin–nicotinamide-induced diabetes was comparable with that of sitagliptin and was slightly lower than that of metformin (13).

A possible explanation for this might be that this compound acts as a strong agonist of GPR119, while the activation of this receptor leads to increasing incretin secretion. Growth of the relative area of insulin-positive endocrine cells and Langerhans pancreatic islet perimeter observed in animals with experimental diabetes mellitus receiving ZB-16 at a dose of 1 mg/kg support the hypothesis that it acts as a protective agent for β-cells. It is also possible that this compound activates the mechanisms of reparative regeneration of β-cells by decreasing the degree of damage and follow-up atrophic changes or by activation of proliferative activity of cambial cells, which was observed in some studies investigating the antidiabetic potential of GRP119 (14, 15).

The abovementioned quantitative changes in the pancreatic tissue as long as improving its function (decreased glycemia level, improved glucose disposal in the glucose tolerance test, and increased glucose-stimulated insulin secretion) indirectly support the hypothesis that ZB-16 compound may stimulate incretin secretion. Further research should be undertaken to investigate the antidiabetic activity of this compound.

## Conclusion

The new GPR119 agonist ZB-16 (1 mg/kg) decreases the blood glucose levels under fasting conditions in animals with the experimental type 2 diabetes mellitus caused by streptozotocin and nicotinamide. The glucose utilization assessed during the glucose tolerance test was also improved by the treatment. The administration of ZB-16 resulted in increase in the relative area of insulin-positive pancreatic endocrine cells. These changes were associated with the increase in stimulated secretion of GLP-1 and insulin, which could be interpreted as the incretin-like action mechanism of ZB-16.

## Ethics Statement

Animal experiments were conducted in accordance with animal research standards defined by Russian law and EASC technical standards for Good laboratory practice (GOST R 53434-2009 and GOST R 51000.4-2011). All procedures were approved by the Regional ethical committee [registration number: IRB 00005839 IORG 0004900 (OHRP)] on February 2, 2014 (protocol number 191-2014).

## Author Contributions

IT and DK designed and supervised the study, and they wrote the manuscript. MC carried out the chemical synthesis and *in vitro* activity evaluation. EV, DB, and EM performed the assessment of hypoglycemic activity and ELISA analysis. AS performed the immunohistochemical assay. EM conducted a statistical analysis of the results.

## Conflict of Interest Statement

The authors certify that they have no potential conflicts of interest connected with publication of the current work.

## Supplementary Material

Figure S1 Correlation between concentrations and agonistic activities of the ZB40-0016 compound and the control substance (Arena) with regard to human GPR119 target receptors. Every concentration point is an average value of two repetitive measurements. ZB40-0016 exhibits nanomolar activity on target GPR119 receptors, indicating that ZB40-0016 is a complete agonist. From RUS Patent 2576037 C1, Russia (2014) (5).
