# Supplementary material for: ZB-16, a Novel GPR119 Agonist, Relieves the Severity of Streptozotocin–Nicotinamide-Induced Diabetes in Rats
Source: Front Endocrinol (Lausanne). 2017 Jul 7;8:152. doi: 10.3389/fendo.2017.00152 (PMC5500613; doi:10.3389/fendo.2017.00152)
Supplement: Figure S1 — Correlation between concentrations and agonistic activities of the ZB40-0016 compound and the control substance (Arena) with regard to human GPR119 target receptors. Every concentration point is an average value of two repetitive measurements. ZB40-0016 exhibits nanomolar activity on target GPR119 receptors, indicating that ZB40-0016 is a complete agonist. From RUS Patent 2576037 C1, Russia (2014) (5). [file Image_1.pdf]

Additional materials

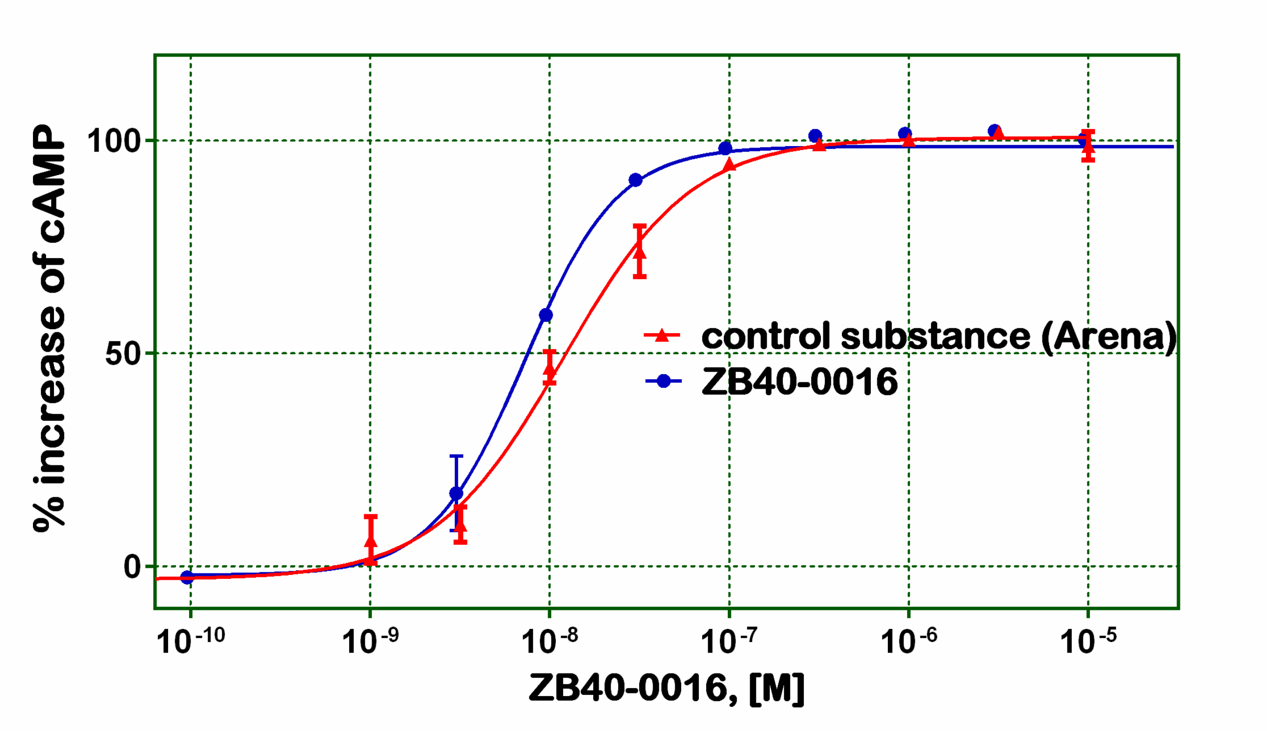

FIGURE S1. Correlation between concentrations and agonistic activities of the ZB40-0016 compound and the control substance (Arena) with regard to hGPR119 target receptors.

Every concentration point is an average value of two repetitive measurements. ZB40-0016 exhibits nanomolar activity on target GPR119 receptors, indicating that ZB40-0016 is a complete agonist. From RUS Patent 2576037 C1. Russia (2014) (5).
